# Association Between Ultra-Processed Food Consumption and Cognitive Performance Among Adolescent Students From Underdeveloped Cities in Brazil: A Cross-Sectional Study

**DOI:** 10.3389/ijph.2024.1607658

**Published:** 2024-10-08

**Authors:** João Victor Laurindo dos Santos, Ingrid Sofia Vieira de Melo, Clara Andrezza Crisóstomo Bezerra Costa, Layanne Cabral de Almeida, Dafiny Rodrigues Silva, Débora Cavalcante Ferro, Déborah Tenório Costa Paula, Mateus de Lima Macena, Nassib Bezerra Bueno

**Affiliations:** ^1^ Faculty of Nutrition, Federal University of Alagoas, Maceió, Brazil; ^2^ Satuba Campus, Federal Institute of Education, Science and Technology of Alagoas, Satuba, Brazil; ^3^ Institute of Chemistry and Biotechnology, Federal University of Alagoas, Maceió, Brazil; ^4^ Paulista School of Medicine, Federal University of São Paulo, São Paulo, Brazil

**Keywords:** adolescent, diet, food intake, ultra-processed foods, cognitive dysfunction

## Abstract

**Objectives:**

The association between ultra-processed foods (UPF) consumption and cognitive performance needs to be better characterized in adolescents, especially in low-income settings, where the cost of human capital is high. This study investigated the association between cognitive performance and UPF in adolescents from the countryside of the Brazilian Northeast.

**Methods:**

Adolescents (15–18 years old) from three public high schools were included. Food intake was assessed using three 24-hour dietary recalls. The classification of foods as UPF was determined according to the Nova classification. Cognitive performance was evaluated using the Non-Verbal General Intelligence Test.

**Results:**

116 adolescents were included, of which 50 (43.1%) showed low cognitive performance. The average energy intake was 1973.5 kcal, with 24.2% coming from UPF. Participants with low cognitive performance consumed 26.5% (95% CI: [22.2; 30.7]%) of daily energy intake from UPF compared to 22.5% ([18.8; 26.2]%) of those with medium-high cognitive performance (*P* = 0.17), without differences in energy and macronutrient intake.

**Conclusion:**

Despite similar UPF consumption compared to the Brazilian average, no association was found between UPF consumption and cognitive performance in this low-income adolescent sample.

## Introduction

Global dietary habits are undergoing a significant transformation, with a concerning increase in the consumption of ultra-processed foods (UPF). In developed countries, these products account for over 50% of total energy intake [1], while in many developing countries, such as those in Latin America, this proportion approaches 30% [2, 3]. Among adolescents in different regions of the world, such consumption varies from 25% to 65% of total daily energy intake [4–6]. According to dietary consumption analysis released in the Brazilian household budget survey of 2017–2018, in general approximately 20% of the Brazilian population’s total daily calories are derived from UPF, and this share reaches 27% in Brazilian adolescents [7]. Factors such as hyper palatability, low cost, easy access, and intense marketing targeted toward the public may explain this trend [8].

The Dietary Guidelines for the Brazilian population, based on the Nova classification, recommends avoiding the consumption of UPF due to its low nutritional composition quality and the use of additives and other components in its fabrication [8, 9]. Excessive intake of UPF was found associated with the development of overweight, obesity, and chronic diseases such as diabetes, hypertension, metabolic syndrome, and dyslipidemia [10]. With the increase in UPF consumption among adolescents, these conditions, which were more prevalent among adult and elderly populations, have become increasingly present in this age group, as well [11]. Moreover, prior studies highlighted the association between UPF consumption and increased risk of cognitive impairment in older people [12, 13], but this association is not clear in adolescents.

It is noteworthy that adolescence represents an important phase of dietary habit formation as well as physical and neural development. Dietary intake during this phase is crucial to ensure the proper development of the prefrontal cortex, which is essential in the process of self-regulation [14]. Therefore, considering the nutritional characteristics of UPF, their consumption may lead to detriments in cognitive development. It is known that the adequate intake of macronutrients and micronutrients contributes to the integrity of the myelin barrier, neural cell membrane, neural proliferation, and synaptic formations [15], potentially impacting the proper cognitive development of individuals. Moreover, high sodium intake is related to cognitive impairment in adult and elderly individuals [16]. The fatty acid profile of the diet also exerts some influence, albeit marginal, with saturated fatty acid consumption being associated with cognitive impairment in adults [17]. In contrast, polyunsaturated fatty acid consumption appears to improve cognitive performance in children [18]. Furthermore, it is not known whether other factors besides the nutritional composition of these foods, such as the processing level and use of non-nutritive additives, may also play a role in the cognitive performance of the individuals [19].

To properly evaluate the impact of diet on cognition, it is essential to clarify the various terms used in this field. Cognitive performance refers to an individual’s ability to perform cognitive tasks that involve mental processes such as memory, attention, executive functions, language, and perception [20, 21]. It is often assessed in clinical and research contexts to understand how different cognitive domains interact and how they can be affected by neurological or psychiatric conditions [20]. Cognitive domains refer to mental processes that involve the acquisition, processing, retention, and use of information [20, 22]. These domains reflect specific areas of mental functioning and include memory, attention, executive functions, language, and perception, among others [20]. Finally, cognitive impairment is defined as a disruption or decline in cognitive functions, indicating that some cognitive abilities are below what is expected for an individual’s age, education, and cultural context [21, 22]. This impairment can be present at any time in a person’s life. It can result from a variety of conditions, such as malnutrition, exposure to heavy metals, metabolic disorders, head trauma, and side effects of drug treatments for conditions such as cancer or Parkinson’s disease [21, 22]. In addition, age-related conditions such as traumatic brain injury, neurodegenerative disorders (such as Alzheimer’s disease), stroke, brain tumors, and brain infections can also cause cognitive impairment [21, 22].

Current studies on the relationship between UPF consumption and cognition are scarce and more common in older adults. A systematic review with meta-analysis of observational studies found associations between higher UPF consumption and cognitive impairment in adults [23]. Others evaluated the association between the consumption of these foods and cognitive performance in adults and elderly individuals in a cross-sectional study, observing a negative relationship between the two [24]. Additional studies corroborated these findings, indicating an association between UPF consumption and cognitive impairment in adults and elderly individuals with type 2 diabetes [13, 25, 26]. Nevertheless, few studies have investigated UPF consumption and cognitive performance in younger populations. Liu et al. conducted a cohort study with 325 Chinese children aged 4–7 years, in which they assessed dietary intake using the Food Frequency Questionnaire and cognitive function using the Verbal Comprehension Index [27]. It was found that children who consumed more than two groups of UPF showed a significant decrease in test scores. However, the consumption was measured by the weekly frequency of consumption of each group of UPF, without mentioning data on daily energy intake from UPF [27].

Thus, studies conducted in populations of different age groups indicate that high consumption of UPF may be associated with inferior cognitive performance. However, this relationship in adolescents, a population in the neural development phase that deserves attention, needs to be better elucidated in the scientific literature. The literature is even scarcer regarding populations in low- and middle-income countries, where the cost of human capital due to inadequate development conditions is even higher. Therefore, this study aimed to evaluate the association between cognitive performance and UPF consumption in adolescents residing in cities in the countryside of the Brazilian Northeast.

## Methods

### Study Design and Ethical Aspects

This study is a secondary cross-sectional analysis of baseline data from a randomized clinical trial called “Internet-Based Nutritional Education versus Conventional Nutritional Education: A Randomized Clinical Trial,” registered in the Brazilian Clinical Trials Registry (ReBEC) under the number RBR-9crqgt. This clinical trial received approval from the Research Ethics Committee of the Universidade Federal de Alagoas, with protocol number 80728017.0.0000.5013, and was conducted in accordance with the principles of the Declaration of Helsinki. All parents or legal guardians of the adolescents provided written consent, and all adolescents consented to participate voluntarily. This article follows the guidelines for Strengthening the Reporting of Observational Studies in Epidemiology-Nutritional Epidemiology [28].

### Population and Sample

Sampling was non-probabilistic, based on convenience. Participants were recruited through invitations made during presentations in the classrooms at three high schools in the state of Alagoas: Escola Estadual Monsenhor Clóvis Duarte de Barros in União dos Palmares municipality; Instituto Federal de Alagoas, in both Murici campus and Satuba Campus, in Murici municipality, and Satuba municipality, respectively. During these presentations, the researchers informed the students about the research and invited them to participate. The state of Alagoas is one of the poorest in the Brazilian Federation, with an average Human Development Index (HDI) of 0.687. It also has the highest illiteracy rate among people aged 15 and older (17.7%), an unemployment rate of 12.0% for individuals aged 14 and older, a GINI index of 0.498, and a significant percentage of households receiving income transfer program benefits: 34.9% from the Bolsa Família Program, 7.1% from the Continuous Cash Benefit program, and 7.3% from other social programs. Additionally, only 34.08% of households are connected to the sewage network, and the general water network supplies 68.14%. At the same time, the studied municipalities have even lower HDIs, with União dos Palmares at 0.593, Murici at 0.527, and Satuba at 0.660 [29, 30].

Adolescents attending these schools, aged between 15 and 18, were included according to the criteria of the World Health Organization (WHO) [31]. Adolescents who presented any condition that prevented anthropometric measurements, those who were HIV-positive, had type 1 diabetes, were pregnant, or were breastfeeding were excluded.

### Variables

#### Exposure

##### Dietary Intake

Dietary intake assessment was conducted through 24-hour dietary recalls administered by trained interviewers. Three 24-hour recalls were collected over three different days, covering two weekdays and one weekend day. During the interviews, participants provided information on foods and beverages consumed from the time they woke up until bedtime. To aid in estimating the quantities consumed, interviewers used two photographic food quantification manuals [32, 33].

The collected data were processed using Avanutri^®^ software, version 4.1 (Avanutri Equipamentos de Saúde Ltda, Rio de Janeiro, Brazil), which converted consumed foods and beverages into energy (kilocalories), carbohydrates, proteins, and fats (g). Information from the following databases was used: Brazilian Food Composition Table [34], Food Composition Table [35], and information provided by food product manufacturers, following this order of preference. Then, foods and beverages were categorized into three subgroups according to the Nova classification: unprocessed and minimally processed foods, processed foods, and UPF [36]. According to the Nova classification, unprocessed foods are obtained directly from plants or animals and undergo no alteration after leaving nature. Minimally processed foods are natural foods subjected to minimal processes such as removal of inedible parts, drying, grinding, filtering, roasting, boiling, pasteurization, refrigeration, freezing, or vacuum packaging, among others. These processes do not add substances to the original food and do not significantly alter its nutritional value. Processed foods are those manufactured by the industry by adding salt, sugar, or other culinary substances to natural or minimally processed foods, aiming to increase durability or improve sensory qualities. UPF are industrial formulations made entirely or largely from substances derived from foods, such as sugars, oils, fats, or salt, and additives like preservatives, antioxidants, and stabilizers. Ultra-processed foods generally contain little or no natural or minimally processed food [36].

### Outcome

#### Non-Verbal General Intelligence Test (NV-GIT)

The NV-GIT assesses non-verbal intelligence and identifies incorrect processes in various types of reasoning. The test consists of 30 multiple-choice questions, each with six response options, only one of which is correct [37]. The reliability and internal consistency of the NV-GIT were verified, and satisfactory results were obtained. The Cronbach’s Alpha coefficient was 0.89, the Spearman-Brown correction was 0.85, and the Test-Retest method showed a coefficient of 0.93. The validity of the NV-GIT was determined through the correlation between NV-GIT scores and four tests: Ravens Progressive Matrices (0.56), R1 (0.42), D70 (0.73), and G36 (0.65) [37].

Developed with the Brazilian population, the NV-GIT is widely used in Brazil. The NV-GIT was chosen for its ease of administration and simple interpretation of results. Unlike other nonverbal intelligence tests, it allows intelligence measures to be expressed on intelligence quotient (IQ) and percentile scales. Additionally, the NV-GIT can assess the mental state of people from 10 to 79 years old, covering three levels of education: Elementary, Secondary, and higher education [37].

The research team, who was previously trained by a psychologist, administered the test to the students. Prior to the test day, the principals from each school were contacted to allow the research team to visit classes, which were paused for 50 min in order for the questionnaires to be administered. All instructions provided in the test manual were strictly followed. There was no time limit, but the test protocols could only be returned after 20 min, as instructed in the test manual, to avoid characterizing withdrawal. Scoring was done using an answer analysis grid and according to the participants’ educational level. Each adolescent was classified into one of the following intelligence level categories [37]: Extremely Low (up to 3 points, IQ below 69), Low (4–7 points, IQ between 70 and 79), Below Average (8–14 points, IQ between 80 and 89), Average (15–22 points, IQ between 90 and 109), Above Average (23–25 points, IQ between 110 and 119), High (26–27 points, IQ between 120 and 129), and Very High (28–30 points, IQ above 130).

For the present analysis, NV-GIT scores were grouped into three main categories to simplify statistical analysis. The scores were categorized as follows: Low cognitive performance, which includes the “Extremely Low,” “Low,” and “Below Average” score ranges; medium cognitive performance, which consists of the “Average” range; and high cognitive performance, which includes the “Above Average,” “High,” and “Very High” ranges.

### Assessment of Covariates

#### Anthropometric Data

For anthropometric assessment, data on weight, height, and Body Mass Index (BMI) were collected. Body weight was recorded using a digital scale (Filizola^®^, São Paulo) with a capacity of 150 kg and a precision of 100g. Height was measured using a portable stadiometer. We used the AnthroPlus software developed by the WHO to assess child growth and calculate z-scores. This software is specifically designed to analyze growth data for children and adolescents aged 5–19 years, allowing comparison with the WHO reference curves. Z-scores were calculated for the following anthropometric indicators: Body Mass Index (BMI) by age and height by age. Participants were categorized according to the WHO reference curves [38]. Waist circumference was measured with a flexible, non-elastic tape positioned at the midpoint between the lower edge of the last rib and the iliac crest [39].

#### Physical Activity Level

Physical activity level was assessed using the short version of the International Physical Activity Questionnaire (IPAQ), validated for the Brazilian population and appropriate for adolescents [40, 41]. This version consists of eight open-ended questions addressing the time spent on activities such as walking, vigorous and moderate physical efforts, as well as periods of physical inactivity (time in a sitting position) during the last week. Based on this information, participants were classified into four levels of physical activity: sedentary, irregularly active, active, and very active.

#### Socioeconomic Level

The socioeconomic level was assessed using the Brazilian Economic Classification Criteria (BECC), developed by the Associação Brasileira de Empresas de Pesquisa (ABEP). Based on the BECC score, participants were classified into six economic classes, ranging from “A”, the highest, to “D-E”, the lowest, taking into account possession of household assets, educational level of the head of the household, and access to services such as piped water and street paving [42].

#### Bias

To avoid bias, three dietary recalls from three different days, comprising two weekdays and one weekend day, were collected. This approach was adopted because the use of three recalls results in more accurate estimates of energy and nutrient intake compared to a lower number of recalls [43, 44].

### Statistical Analyses

Continuous variables were reported using the mean and the standard deviation measures, and categorical variables were exhibited as relative and absolute frequencies. Comparisons between groups for continuous variables were made using a one-way analysis of variance (ANOVA), whereas the chi-square test was utilized for categorical variables. To explore the relationship between low cognitive performance and ultra-processed food consumption, an analysis of covariance (ANCOVA) was used, adjusted for sex, age, body mass index, socioeconomic status, and physical activity level. The estimated marginal means (EMM) for energy intake according to food processing level, in percent, were calculated. An alpha value of 5% was adopted for all analyses. JAMOVI software version 4.2.0 (Sydney, Australia) was employed for all analyses.

## Results

**FIGURE 1:**
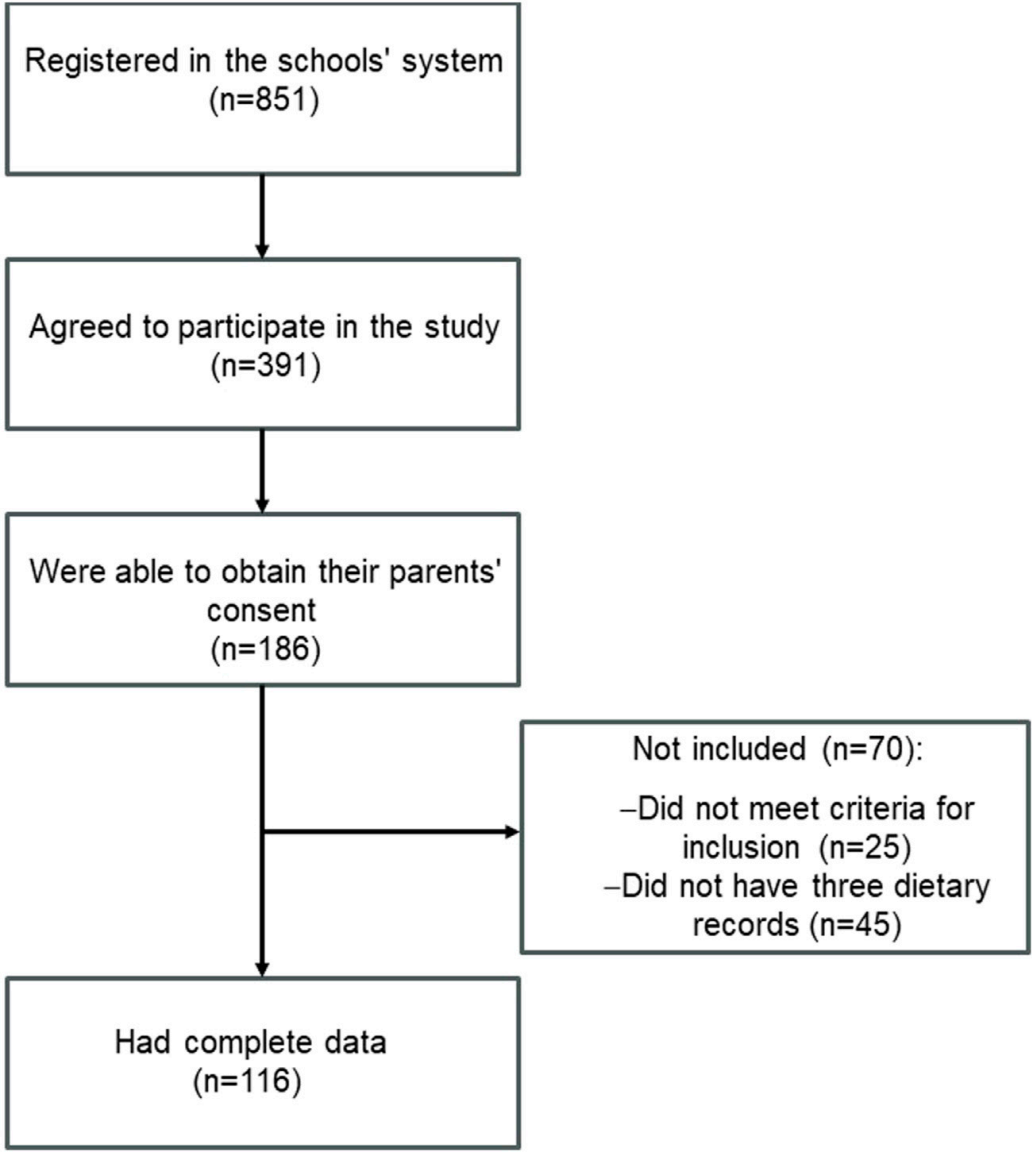
Flowchart of participant selection (Brazil, 2018–2019).

Within the age range of interest for our study, there were a total of 851 potentially eligible students from the three schools. From these, 186 were interested in participating and were able to provide their parents’ consent, and hence, they were recruited. After excluding 25 participants due to eligibility criteria and 45 participants who did not complete three dietary recalls, the final sample consisted of 116 adolescents, as shown in Figure 1. The participants had a mean age of 16.7 (0.9) years and a mean BMI-for-age of 0.02 (1.07) Z-score. Among the included adolescents, the classification based on raw NV-GIT scores was as follows: Extremely Low (n = 4; 3.4%), Low (n = 17; 14.7%), Below Average (n = 29; 25.0%), Average (n = 45; 38.8%), Above Average (n = 15; 12.9%), High (n = 4; 3.4%), and Very High (n = 2; 1.7%). Specifically, as categorized for the present analysis, 50 (43.1%) had a low, 45 (38.8%) had a medium, and 21 (18.1%) had a high non-verbal intelligence level. The socioeconomic and anthropometric characteristics of the participants are presented in Table 1. No statistically significant differences were observed for variables such as age, weight, height, or waist circumference across the cognitive performance categories. However, the BMI-for-age Z-score showed a statistically significant difference (*p* = 0.01), although this difference was not clinically relevant, as the values remained within normal growth ranges for adolescents. Additionally, no significant differences were found in the level of physical activity (assessed by the IPAQ) or in socioeconomic classification (BECC) across the cognitive performance categories.

**TABLE 1:** Sociodemographic characteristics of the sample (n = 116) (Brazil, 2018–2019).

Variables	Total sample (n = 116)	(NV-GIT)			
		Mean (SD) Low (n = 50; 43.1%)	Medium (n = 45; 38.8%)	High (n = 21; 18.1%)	*p*-value^a^
Age (years)	16.65 (0.86)	16.86 (0.78)	16.47 (0.87)	16.52 (0.98)	0.06
Weight (kg)	60.69 (12.12)	59.26 (10.30)	62.56 (14.60)	60.10 (10.14)	0.40
Height (m)	1.66 (0.09)	1.67 (0.10)	1.65 (0.09)	1.71 (0.10)	0.09
WC (cm)	72.08 (8.22)	70.68 (7.63)	73.41 (9.06)	72.60 (7.53)	0.26
BMI-for-Age (Z-score)	0.02 (1.07)	0.13 (1.03)	0.37 (1.06)	−0.35 (1.05)	0.01
	n (%)	n (%)	n (%)	n (%)	*p*-value^b^
BECC					0.07
A, B1 and B2	23 (19.8)	8 (16.0)	7 (15.6)	8 (38.1)	
C1, C2 and D-E	93 (80.2)	42 (84.0)	38 (84.4)	13 (61.9)	
IPAQ					0.09
Very active and active	78 (67.2)	33 (66.0)	30 (66.7)	15 (71.4)	
Irregularly active and sedentary	38 (32.8)	17 (34.0)	15 (33.3)	6 (28.6)	

BECC, Brazilian economic classification criteria; WC, waist circumference; W-A, Weight-for-age; BMI-A, Body mass index-for-age; IPAQ, international physical activity questionnaire; NV-GIT, Non-verbal general intelligence test.

^a^ *p*-value for ANOVA one-way.

^b^ *p*-value for chi-square tests.

Table 2 presents the dietary intake analysis. The mean energy intake of the sample from the three dietary recalls of each individual was 1973.5 (711.6) kcal, with a mean UPF intake of 503.6 (14.9) kcal, for a mean dietary UPF share of 24.2 (14.9) %. It is noteworthy that there were no differences in dietary energy intake and macronutrient content across levels of NV-GIT.

**TABLE 2:** Dietary characteristics of the sample (n = 116) (Brazil, 2018–2019).

Variables	Total sample (n = 116)	(NV-GIT)			
		Low (n = 50; 43.1%)	Medium (n = 45; 38.8%)	High (n = 21; 18.1%)	*p*-value^a^
	Mean (SD)	Mean (SD)	Mean (SD)	Mean (SD)	
Total energy (kcal)	1,973.56 (711.67)	2,016.97 (804.00)	1,881.05 (659.96)	2,068.47 (580.48)	0.61
Carbohydrate (%)	52.68 (7.96)	53.00 (9.21)	51.74 (6.83)	53.84 (6.68)	0.49
Protein (%)	20.12 (7.33)	20.84 (9.52)	20.03 (4.93)	18.42 (4.21)	0.74
Lipids (%)	27.69 (5.29)	27.33 (5.01)	28.22 (6.03)	27.58 (4.55)	0.74
Fiber (g)	21.77 (10.59)	20.92 (10.44)	22.41 (11.93)	22.74 (8.07)	0.52
Unprocessed or Minimally Processed (kcal)	976.95 (396.19)	957.15 (373.28)	986.74 (433.36)	1,003.13 (382.29)	0.88
Unprocessed or Minimally Processed (%)	50.90 (16.33)	50.40 (17.34)	52.20 (15.50)	49.35 (16.17)	0.23
Processed (kcal)	492.92 (302.10)	467.23 (309.13)	474.17 (300.22)	594.30 (281.33)	0.11
Processed (%)	24.82 (12.47)	23.03 (12.41)	25.12 (12.26)	28.48 (12.87)	0.77
Ultra-processed (kcal)	503.68 (407.59)	592.59 (492.03)	420.15 (282.25)	471.04 (387.67)	0.24
Ultra-processed (%)	24.26 (14.89)	26.57 (16.12)	22.67 (13.59)	22.17 (14.48)	0.34

^a^ *p*-value for ANOVA one-way.

Table 3 presents estimated marginal means for energy intake, in percent, according to the food processing level in the different classes of nonverbal intelligence level. No significant associations were identified between these variables. To further explore our data, we have merged the groups with medium and high cognitive performance and tested their percent of UPF intake against those of the low cognitive performance group. Although the statistical power increased, there were no significant differences between groups (EMM of %UPF intake for low cognitive performance: 26.5 [22.2; 30.7]%; medium-high performance: 22.5 [18.8; 26.2]%; *P* = 0.17).

**TABLE 3:** Estimated marginal means of multivariable analysis between Non-Verbal General Intelligence Test (NV-GIT) classifications and percentage of foods according to Nova (n = 116) (Brazil, 2018–2019).

Food intake	(NV-GIT)			
	Low	Medium	High	*p*-value^a^
	EMM [CI 95%]	EMM [CI 95%]	EMM [CI 95%]	
Unprocessed or Minimally Processed (%)	49.84 [45.17; 54.51]	52.52 [47.60; 57.44]	49.97 [42.67; 57.27]	0.71
Processed (%)	23.42 [19.86; 26.98]	25.05 [21.31; 28.80]	27.67 [22.12; 33.23]	0.44
Ultra-processed (%)	26.73 [22.47; 30.98]	22.41 [17.93; 26.89]	22.35 [15.70; 28.99]	0.32

^a^ Analysis of covariance adjusted for sex (female and male), age (years), body mass index-for-age (Z-score), Brazil Economic Classification Criterion (A, B1 and B2, and C1, C2, D-E), and International Physical Activity Questionnaire (Very active and active, and irregularly active and sedentary).

As an exploratory analysis, we tested which exposures were associated with the higher intake of UPF in our sample. Nevertheless, neither sex, excess weight, BBEC, nor IPAQ showed significant associations with the dietary share of UPF intake (data not shown).

## Discussion

In the present study, the NV-GIT tool revealed a high prevalence of low cognitive performance (43.1%) among adolescents enrolled in three public schools in the interior of the state of Alagoas, Brazil. On average, 24.2% of their energy intake was derived from UPF, and this consumption was not associated with cognitive performance, according to the NV-GIT tool. The individuals with low cognitive performance also showed the same level of macronutrient intake, unprocessed and minimally processed foods as the individuals with medium and high cognitive performance, indicating that, in the present sample, no measures of dietary intake were associated with the degree of cognitive performance.

The lack of a significant association between UPF consumption and performance on the NV-GIT in the present study may be explained by the specifics of the cognitive aspects assessed by this test. The NV-GIT focuses on non-verbal cognitive skills such as problem-solving, visual-spatial perception, and abstract reasoning. These aspects may be less sensitive to the potential influences of UPF consumption compared to other cognitive skills. Previous studies with adults and children have identified an association between UPF consumption and cognitive function [13, 24, 27]. In these studies, cognitive function was assessed through tests that measured both verbal and non-verbal skills, whereas the NV-GIT focuses only on non-verbal skills. Additionally, there may be a significant difference between verbal and non-verbal skills in low-income participants, with a higher overall average in the non-verbal skills test [45], like those included in this research. This disparity can influence the results of cognitive tests differently. When we decided to use a non-verbal intelligence test, we assumed that it would be possible to identify students with low cognitive performance more accurately since the overall average of these tests is high in this age range. Those with lower averages could represent individuals with more significant cognitive difficulties. This approach is particularly relevant for low-income students, who may show a big gap between their verbal and non-verbal scores [45].

The heterogeneity found in the published studies hampers our ability to compare our prevalence of low cognitive performance with the literature. Few of the published studies report such prevalence rates, and they usually use different tools to assess cognitive performance. In many of these studies, only the mean scores obtained in the various cognitive domains are reported [13, 24, 26]. Furthermore, such limitation also extends to the comparison of the findings regarding the specific association between UPF consumption and cognitive performance in adolescents, with only studies relating dietary patterns to cognitive performance in this age range [46–50]. Future studies should investigate a wider number of cognitive domains in order to assess the potential association of UPF intake with this complex outcome, especially in adolescents.

Our hypothesis that UPF intake could negatively impact cognition in adolescents was supported by the unique nutritional characteristics of UPF. These foods tend to be rich in added sugars and saturated fats, factors that have been associated with effects that may contribute to cognitive deficits [17, 19, 26, 51–53]. Increasing evidence also indicates that the consumption of UPF is associated with a higher intake of pro-inflammatory ingredients and oxidative stress, both recognized as contributing factors to cognitive impairment [54–57]. Furthermore, UPF consumption may disrupt the gut microbiota, leading to an imbalance known as gut dysbiosis, which is associated with a higher risk of cognitive impairment [58–61]. Additionally, obesity-induced adiposity, often related to excessive UPF consumption, is marked by the presence of pro-inflammatory cytokines, which have been associated as contributing factors to cognitive impairment [62–65]. In contrast, unprocessed and minimally processed foods are rich in beneficial nutrients, such as essential fatty acids, polyphenols, and vitamins, with well-established antioxidant effects [66, 67]. Studies have shown that adopting healthy dietary patterns, which include a variety of these foods, can contribute to better cognitive functioning, even promoting structural changes in the brain, such as a larger left hippocampal volume, white matter, and gray matter [68–70]. These brain structures are associated with better cognitive functioning [71–73].

It is noteworthy that the share of dietary UPF consumption among the adolescents in our research was similar to the Brazilian adolescents’ mean of 27.0%, as reported by the Brazilian household budget survey of 2017–2018 [7]. Also, other studies conducted with Brazilian adolescents show similar results. Rocha et al. found that UPF contributed an average of 28% of the total energy intake among adolescents in Brazilian public and private schools [74], and Martins et al. investigating adolescents from the same Brazilian region also found a share of 26% of energy arising from UPF [75]. It is noteworthy that this share of dietary energy arising from UPF is way lower compared to studies conducted in developed countries, such as the United States and Australia, where adolescents had a share of dietary energy arising from UPF of 67.0% and 54.3%, respectively [76, 77].

This study has some limitations. First, it is a cross-sectional study, which prevents the determination of causality between the investigated variables. Another limitation of the study was the use of a questionnaire to assess participants’ physical activity level, which may also be subject to memory bias, overestimation or underestimation of physical activity, and difficulty in determining the intensity of the activity. Additionally, the sample size was modest, which may affect the generalizability of the findings and the low statistical power of the study. It is worth noting that our study has some significant strengths. The analysis was conducted on an age group that is less studied, and most studies on the topic are conducted in developed countries, which helps fill knowledge gaps in this specific population. Another strength was the use of three 24-hour recalls, which provided a more accurate estimate of the participants’ dietary intake [43, 44]. Additionally, the study made adjustments for relevant confounding factors, thereby improving the understanding of the analytical framework.

In conclusion, UPF consumption was not associated with cognitive performance in the NV-GIT in adolescents residing in underdeveloped cities in Brazil. Although the nature of our study does not establish direct causal relationships and the current sample was not specifically recruited to test such hypotheses, the results can be viewed as exploratory and suggest that the potential role of consuming these foods on the cognitive performance of adolescents deserves further investigation, especially for different cognitive domains.
